# Anterior cruciate ligament reconstruction, can an anatomic femoral tunnel be achieved with the trans-tibial technique? Cadaveric study

**DOI:** 10.1186/s40634-021-00444-w

**Published:** 2022-01-10

**Authors:** Daniel Acevedo Tobler, Santiago Hermosilla, Natalia Otero, Juan Kenny, Juan Del Castillo

**Affiliations:** 1 Cátedra de Traumatología y Ortopedia, Universidad de Montevideo, Montevideo, Uruguay; 2 Departamento de Anatomía, Facultad de Medicina Universidad de la República, Montevideo, Uruguay; 3 Clínica de Traumatología y Ortopedia, Facultad de Medicina Universidad de la República, Montevideo, Uruguay

**Keywords:** Anterior cruciate ligament, Reconstruction, Trans-tibial tunnel, Femoral tunnel

## Abstract

**Purpose:**

To evaluate the possibility to access the anatomic femoral insertion of the anterior cruciate ligament (ACL) through trans-tibial (TT) and trans-portal technique, for ACL reconstruction in an independent way. To register anatomical characteristics of the TT tunnels.

**Methods:**

Ten formaldehyde preserved knee anatomic articular specimens were dissected. Femoral tunnels were confectioned reproducing the original topography of the ACL. First, the femoral tunnel was made with the independent trans-portal technique. Then, utilizing the tibial stump of the ACL and tibial guides at 45°, the TT tunnels were confectioned trying to match the previously made femoral tunnel by trans-portal technique.

**Results:**

In all specimens, match between the TT tunnel with the independent trans-portal tunnel was achieved. Mean values for TT coronal angle was 53,0°, for transversal angle 43,3° and for distance from tunnel to joint line 2,55 cm. A horizontalization and medialization of the TT tunnels had to be made to adequately match with the femoral tunnel made by the independent trans-portal technique.

**Conclusions:**

By macroscopic anatomic and independent means, an anatomic femoral tunnel was confectioned with the TT technique matching with the anatomic femoral tunnel made in an independent way. As clinical relevance, the present study allows to anatomically assess the possibility to perform an anatomic femoral tunnel through the TT technique.

**Level of evidence:**

V

## Introduction

Anterior cruciate ligament (ACL) rupture is a frequent lesion, with an incidence of 68,6 per100.000/ year, with an important sport association. The incidence varies according to the age and sex of the patient, which could reflect different stages of exposition to different sport activities, whereas the increase in the reconstructive surgery can be a manifest of a higher wish of the patients to retake high demand activities after a ACL lesion [8].

Approximately, 300.000 ACL reconstructions are made in the United States in one year. Without its proper treatment, this lesion results in an increment of articular laxitude, knee instability, biomechanical disturbance, reduced physical activity and decrease in sport participation [4].

A key step in the reconstruction is the emplacement of the bone tunnels for the graft, particularly it has been noted that a wrong positioning of the femoral tunnel is one of the most frequent failure causes.

In recent years, techniques that achieve a femoral tunnel that imitates the original ACL insertion topography have gained popularity, as well as performing independent femoral and tibial tunnels by the antero-medial portal (AMP) or by out-in technique. The TT techniques had been catalogued as non-anatomic, for which modifications had been coming to light to achieve a more anatomical location of the tunnels.

Multiple studies had been made showing the differences and similitudes between independent femoral tunnel techniques and TT tunnel, concluding that, clinically, there are no statistically significant differences between both techniques [3, 4, 7, 9, 10]. Although it is clearly demonstrated in biomechanical laboratory studies that reconstructions with anatomic techniques provide better control of anterior translation and rotation motion of the knee. Nevertheless, in some series the graft failure rate is even higher in anatomical reconstructions.

Actually, both techniques are scientifically accepted and show excellent clinical results [4]. Anatomical studies allow us to compare both techniques with a relevant clinical application.

## Methods

Ten articular anatomic human knee specimens were used, from Departamento de Anatomía de la Facultad de Medicina de la República Oriental del Uruguay, six male gender, four female gender, preserved with formaldehyde 10% that did not have any previous incision and no morphologic alteration.

As exclusion criteria, advanced arthrosis or absence of the ACL were proposed.

**Fig. 1:**
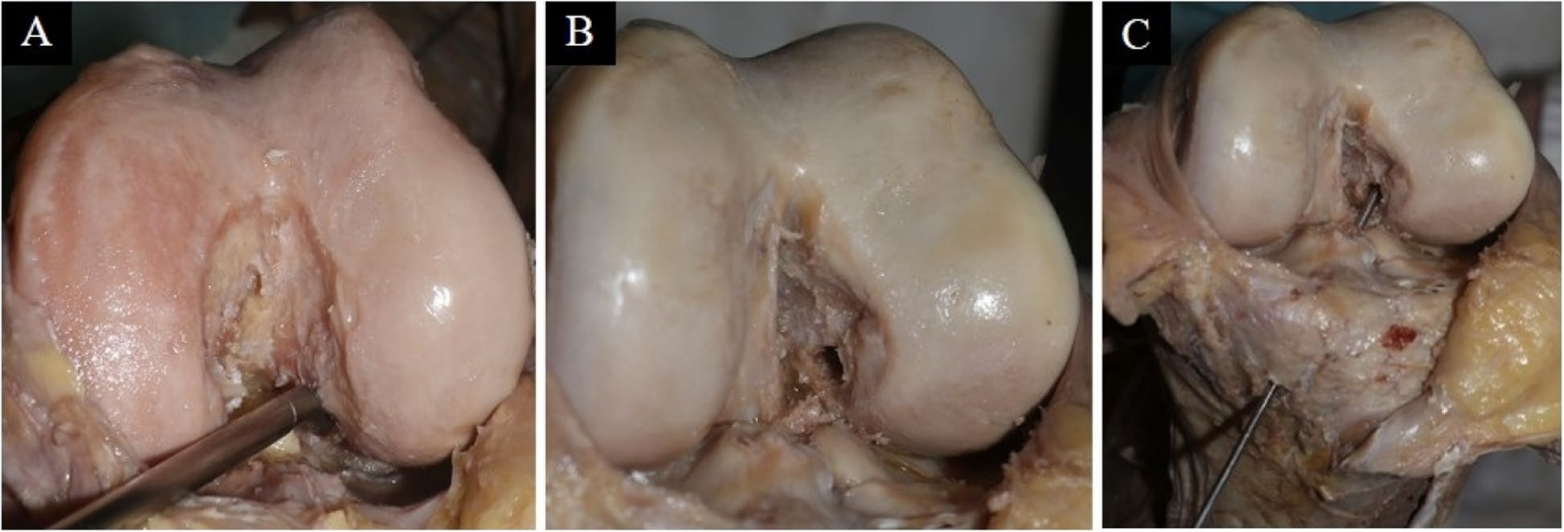
Femoral tunnel confection. **A.** Femoral tunnel performed by anteromedial portal. **B.** Femoral tunnel already performed. **C.** Matching of tibial tunnel with femoral tunnel (non-anatomical technique)

A medial incision was made with detachment of the patellar tendon from the anterior tibia tuberosity (ATT). Tibia and femoral guide from ACL, and drill bits of 8 mm were used to make the tunnels. ACL section was performed in each preparation, leaving both the tibial and femoral insertion stumps. Next, in each preparation, the tunnel was made with the independent AMP in anatomical position, over the ACL femoral stump, locating the guide in the area that best mimic the posterolateral fascicle of the ACL. 8 mm drill bits were used and a 25 mm tunnel was achieved with 120° flexion of the knee. Later, in the same specimen, with a 90° flexion, the tibia tunnel was made with tibial guide at 45°, pre-established sagittal plane angle for the postero-lateral fascicle reconstruction [4], using a guide wire from the previously performed femoral tunnel to the tibial insertion of the ACL (Fig. 1).

In this way, we intend to match the tibial tunnels as if using the TT technique, with the femoral tunnels previously made by the anatomical AMP technique.

Anatomic characteristics of the tibia tunnels interest were registered; the angle in the coronal plane centered over the ATT and the center of the tibia tunnel entry orifice (CATATT); the angle in the transversal plane centered over the ATT and the tibial tunnel entry orifice; finally, the distance from the center of the tibia tunnel entry orifice to the tibia plateau border was measured (DTTP).

## Results

In all specimens, it was possible to achieve an anatomical femoral entry with the TT technique, matching with the previously femoral tunnel made through independent anatomical technique.

**Fig. 2:**
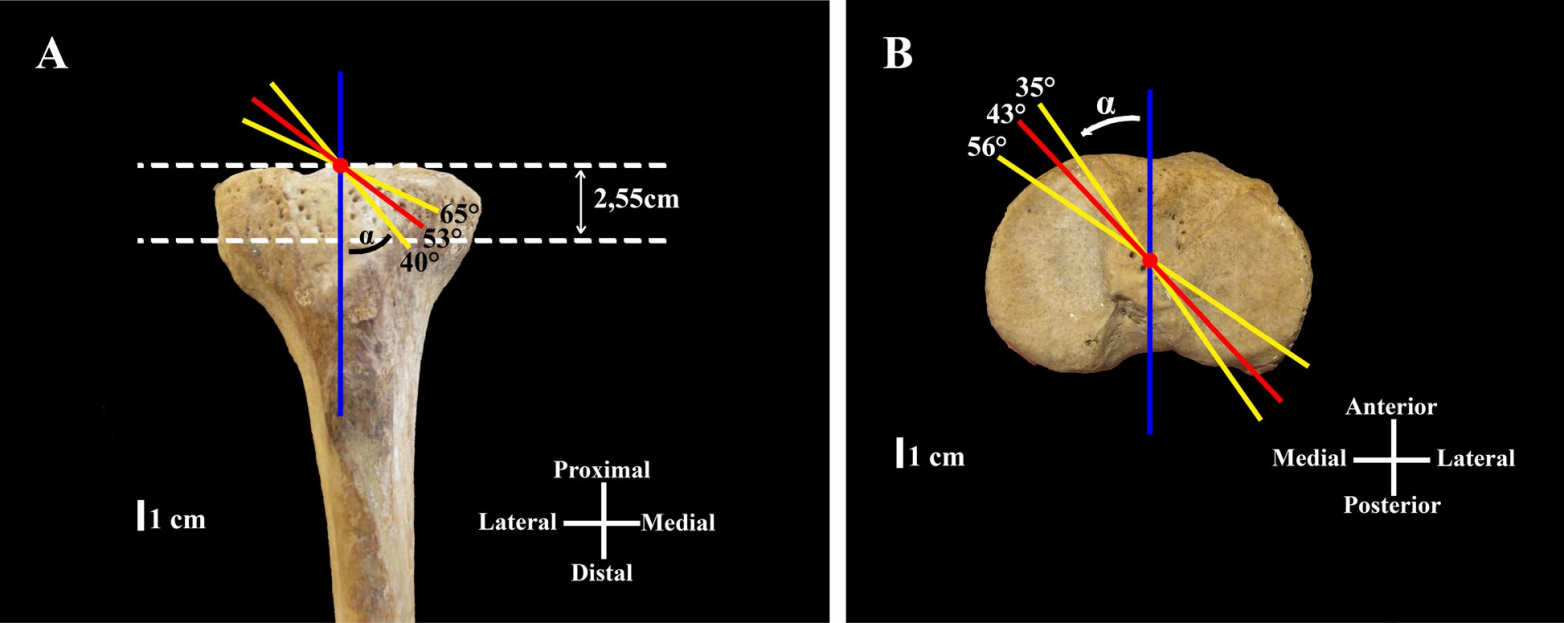
Schematic representation of recorded measurements on dry bone. **A**. Coronal angle (CATATT) minimum, maximum, and mean value, tunnel-plateau distance (DTTP) mean. **B**. Transverse angle (TATATT) minimum, maximum and mean value

CATATT, TATATT angular values and DTTP distance for each dissection are presented in Table 1. Mean values were 53,0° and 43,3° for CATATT and TATATT angles, and mean distance DTTP was 2,55 cm (Fig. 2).

**Table 1:** Coronal and transverse angle, and tunnel-plateau distance for each dissection

Dissection ID number	Coronal Angle (CATATT)	Transverse Angle (TATATT)	Distance Tibial Tunnel-Plateau (DTTP)
**1**	55	36	2,5 cm
**2**	60	40	2,0 cm
**3**	45	45	3,0 cm
**4**	50	45	3,0 cm
**5**	60	44	2,0 cm
**6**	40	37	3,0 cm
**7**	65	55	2,0 cm
**8**	55	56	2,5 cm
**9**	45	40	3,0 cm
**10**	55	35	2,5 cm

Measurements were performed with a transparent 360^0^ 12′ goniometer (EGM-429) in coronal and axial planes with reference to the tibial guide wire inserted.

## Discussion

The most important finding of this study is that it was possible to achieve an anatomical femoral entry with the TT technique, matching the previously femoral tunnel made through independent anatomical technique.

The concept of anatomical and individualized treatment of ACL reconstruction refers to trying to recreate as faithfully as possible the individual anatomy of the patient in order to imitate the native characteristics of the ACL and improve patient outcomes (3; 9; 10). The concept of anatomical reconstruction is based on restoring the 2 fascicles functionally, obtaining previous native insertions of the ACL, correctly tensioning each fascicle and individualizing the surgery for each patient [4]. This is important considering the high individual variability of the ACL anatomy. In one study, the anatomical characteristics of the ACL were recorded with *n* = 16 (femoral insertion length, thickness and area) resulting in differences between 30% and 40% in these variables [2]. Knowledge of the anatomy of the ACL is the cornerstone of the steps in these techniques. ACL presents 2 fascicles, one postero-lateral (PL) and the other antero-medial (AM). The ACL has a tibial insertion at the level of the anterior intercondylar area of the tibia and a femoral insertion at the level of the lateral intercondylar crest, which in turn is divided by the lateral bifurcated ridge, perpendicular to the latter, which defines the areas of insertion of these two issues, the AM and the PL [3, 9, 10].

The tibial insertion has a fan-out shape with an average insertion length of 17 mm in the sagittal plane and 11 mm of average width in the coronal plane. This insertion is aligned with the anterior horn of the lateral meniscus and is closely related to the tibial spines. The femoral insertion is oval in shape, smaller than the tibial insertion [4, 6].

Certain studies have suggested the ideal location for the tibial tunnel. For Morgan, the external orifice of the tibial tunnel should be 1 cm superior to the superior edge of the pes anserine and 1.5 cm posteromedial with respect to its superior margin [3, 9, 10].

The ideal tunnel is 4-5 cm long. A short tunnel can lead to two problems: an oblique path with an oblong exit hole leading to elongation; that its position is much anterior, which leads to impingement of the intercondylar roof, loosening of the graft and loss of flexion. For Hulet, the tunnel must be 25° oblique in the frontal plane. In the transverse and sagittal plane it should be between 40 and 60° for this author [3, 9, 10].

Its orientation is important, since its horizontalization leads to a larger intra-articular orifice. This results in little contact between the graft and the tunnel, with greater mobility of the graft and a greater risk of failure [3, 9, 10].

**Fig. 3:**
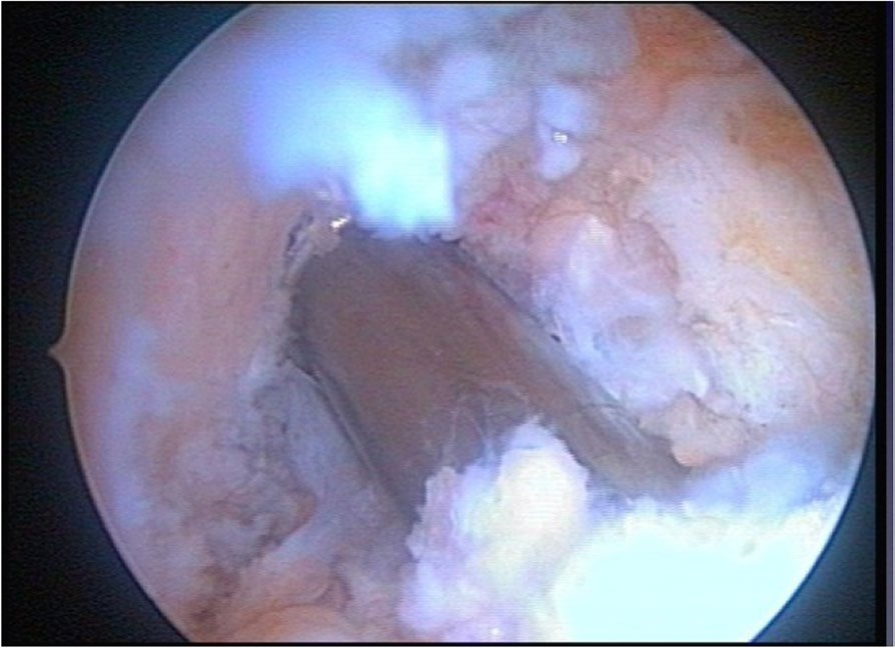
Femoral tunnel made with TT technique, arthroscopic view

There are 2 types of techniques for making the tunnels: the non-anatomical or dependent and the anatomical or independent. The trans-tibial technique (Fig. 3) belongs to the first group, while the AMP technique, outside-in and inside-out technique with reverse drilling, belongs to the second group [4].

The trans-tibial technique is called non-anatomical because of the discrepancy between the position of the femoral tunnel and the native insertion of the ACL. Therefore, independent techniques have gained popularity. Each of these has its advantages and disadvantages [4].

Certain studies have shown greater failure with the antero-medial technique (5.16%) than with the trans-tibial technique (3.20%). One explanation for this is that, since the graft is not in anatomical position, it is subjected to less stress [4].

The technique to be used should be based on a combination of variables, including experience, equipment, cost, efficiency, age of the patient, patient activity, type of graft, and cosmetics, since recent systematic reviews and guidelines do not support any technique above one another, because the results are similar [4].

In the TT technique, the femoral tunnel is dependent on the exit of the tibial tunnel. A low position femoral tunnel that mimics the PL bundle would theoretically repair a less effective ligament in relation to knee stability [5].

In the trans-tibial technique, one uses to alternate the degrees of knee flexion to perform the femoral tunnel and the transverse angle of the drill. Safety ranges have been defined. With a knee in 120 ° flexion and with a maximum transverse reaming angulation of 10 °, they could be recommended to prevent injury to lateral femoral structures (lateral collateral ligament, lateral epicondyle, lateral head of gastrocnemius) when the femoral tunnel is performed at through the trans-tibial technique [1].

The AM technique allows more anatomical precise location when tunneling and reestablishing native relationships of attachments. Although non-anatomical techniques result in measurable kinematic changes (rotationally and in translation), the clinical results have not shown differences between these two techniques [7].

Regarding the surgical technique, extra articular guides adjusted to 55 ° are recommended to place the pin [3, 9, 10]. This can vary depending on whether you want to perform a single fascicle reconstruction, in which case the guide is placed at 55 °; or if you want to perform a reconstruction of two fascicles, where a 45 ° angle of the guide would be made for the PL fascicle and a 55 ° angle for the AL [4]. Neither of these techniques has shown superiority over the other [6].

A beneficial role of preserving ACL remnants has been proposed, as this may facilitate proper placement of the guides as an anatomical reperfusion. In addition, these remnants have biomechanically functional fibers that protect the graft, contribute to the proprioception and vascularization of the graft [4].

In our series, the femoral tunnel made by the AM portal could be reached always through the tibial tunnel, but it demanded a change in tunnel direction. It had to be done from a more proximal and more medialized starting point in the tibia, thus horizontalizing the tunnel.

As we saw in our specimens, when trying to make a tibial tunnel that allows us to reach an anatomical femoral tunnel in a TT way, the more medial we go with the guide, the greater the TATATT and CATATT we need, shortening the DTTP.

The horizontalization of the tunnel also causes the articular orifice of the tibial tunnel to undergo modifications, leaving it more ovoid and occupying more space on the inter-spinous surface, this can generate that the articular exit of the tunnel may be more posterior than desired, which can lead to residual instability.

**Fig. 4:**
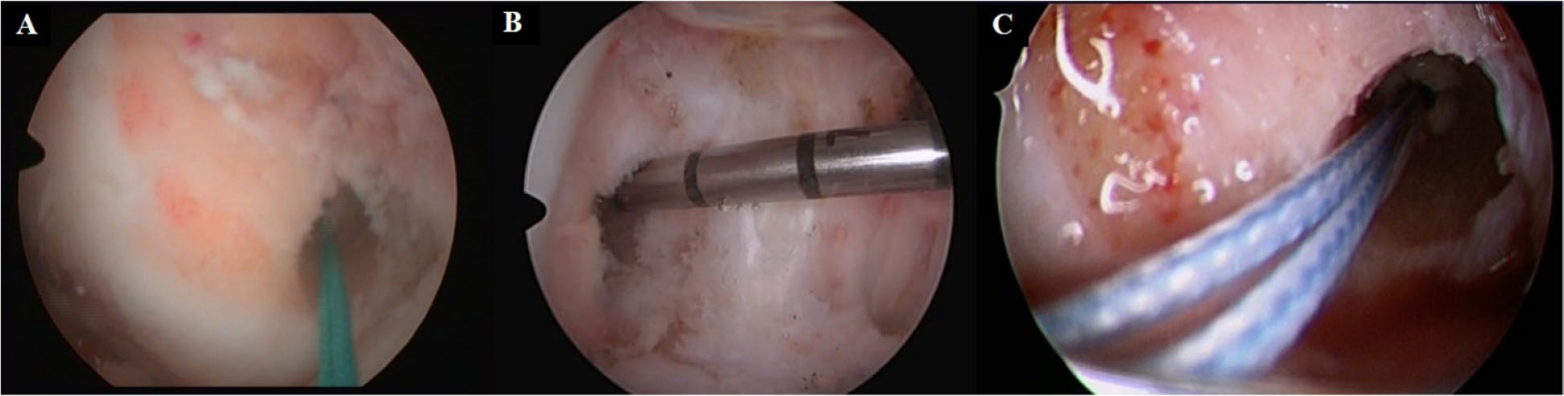
Anatomic femoral tunnels in 3 patients (**A**), (**B**), (**C**), arthroscopic view

In in-vivo situations (Fig. 4), to be more anatomical in the trans-tibial technique, it requires to be performed in two stages, placing the guide wire eccentrically in the tunnel and to perform the femoral tunnel, the tibial tunnel is overdriven, this alters its three-dimensional shape from a cylinder to an hourglass. These irregularities can lead to failure of graft fixation.

## Conclusions

Our anatomical study shows that it is possible to perform an anatomical femoral tunnel using both the trans-portal and trans-tibial techniques, but it demands a different, more horizontal, direction in the tibial tunnel.

When we seek to be anatomical when performing the femoral tunnel by modifying the trans tibial technique, we see that we can alter the exit of the tibial tunnel in the inter-spinous space, running the risk of being less anatomical at the tibial level. Therefore, the independence of the tunnels appears to be an advantage for the anatomical location of both tunnels.

The use of cadaveric specimens as well as a rigorous knowledge of ACL anatomy, allows us to correlate both techniques to assess the advantages and disadvantages of these ACL reconstruction techniques.
